# Rescue pulmonary vein isolation for hemodynamically unstable atrial fibrillation storm in a patient with an acute extensive myocardial infarction

**DOI:** 10.1186/1471-2261-12-110

**Published:** 2012-11-26

**Authors:** Itsuro Morishima, Takahito Sone, Hideyuki Tsuboi, Hiroaki Mukawa

**Affiliations:** 1 Department of Cardiology, Ogaki Municipal Hospital, 4 -86 Minaminokawa-cho, Ogaki, 503-0864, Japan

**Keywords:** Atrial fibrillation, Acute myocardial infarction, Cardiogenic shock, Catheter ablation, Congestive heart failure, Pulmonary vein isolation

## Abstract

**Background:**

New-onset atrial fibrillation in patients hospitalized for an acute myocardial infarction often leads to hemodynamic deterioration and has serious adverse prognostic implications; mortality is particularly high in patients with congestive heart failure and/or a reduced left ventricular ejection fraction. The mechanism of atrial fibrillation in the context of an acute myocardial infarction has not been well characterized and an effective treatment other than optimal medical therapy and mechanical hemodynamic support are expected.

**Case presentation:**

A 71 year-old male with an acute myocardial infarction due to an occlusion of the left main coronary artery was treated with percutaneous coronary intervention. He had developed severe congestive heart failure with a left ventricular ejection fraction of 34%. The systemic circulation was maintained with an intraaortic balloon pump, continuous hemodiafiltration, and mechanical ventilation until atrial fibrillation occurred on day 3 which immediately led to cardiogenic shock. Because atrial fibrillation was refractory to intravenous amiodarone, beta-blockers, and a total of 15 electrical cardioversions, the patient underwent emergent radiofrequency catheter ablation on day 4. Soon after electrical cardioversion, ectopies from the right superior pulmonary vein triggered the initiation of atrial fibrillation. The right pulmonary veins were isolated during atrial fibrillation. Again, atrial fibrillation was electrically cardioverted, then, sinus rhythm was restored. Subsequently, the left pulmonary veins were isolated. The stabilization of the hemodynamics was successfully achieved with an increase in the blood pressure and urine volume. Hemodiafiltration and amiodarone were discontinued. The patient had been free from atrial fibrillation recurrence until he suddenly died due to ventricular fibrillation on day 9.

**Conclusions:**

To the best of our knowledge, this is the first report of pulmonary vein isolation for a rescue purpose applied in a patient with hemodymically unstable atrial fibrillation complicated with an acute myocardial infarction. This case demonstrates that ectopic activity in the pulmonary veins may be responsible for triggering atrial fibrillation in the critical setting of an acute myocardial infarction and thus pulmonary vein isolation could be an effective therapeutic option.

## Background

New-onset atrial fibrillation (AF) in patients hospitalized for an acute myocardial infarction (AMI) often leads to hemodynamic deterioration and has serious adverse prognostic implications; mortality is particularly high in patients with congestive heart failure and/or a reduced left ventricular (LV) ejection fraction [1,2]. The mechanism of AF in the context of an AMI has not been well characterized, and little has been published on the management strategies for AF in this setting.

## Case presentation

**Figure 1:**
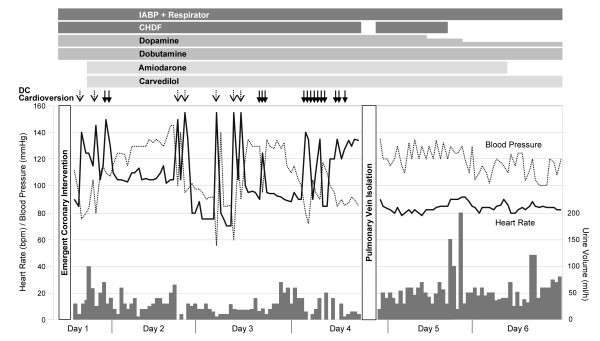
**Changes in hemodynamics before and after pulmonary vein isolation.** Atrial fibrillation led to deterioration of hemodynamics as shown by the decrease in blood pressure and in urine volume. Pulmonary vein isolation restored sinus rhythm and successfully improved hemodynamics. Solid arrows indicate electrical cardioversion to terminate atrial fibrillation; dotted arrows indicate electrical cardioversion to terminate ventricular tachycardia. CHDF = continuous hemodiafiltration; IABP = intraaortic balloon pump.

A 71-year-old male with an AMI due to a total occlusion of the left main coronary artery was treated with emergent percutaneous coronary intervention. The patient had no history of AF and was in sinus rhythm on admission to the coronary care unit. He had developed severe congestive heart failure and was on mechanical ventilation under intravenous sedation with midazolam. Echocardiography revealed an LV ejection fraction of 34% and a left atrial (LA) diameter of 46 mm. The systemic circulation was maintained with an intravenous administration of inotropes, an intraaortic balloon pump (IABP), and continuous hemodiafiltration (CHDF). Soon after admission, the patient had several episodes of ventricular tachycardia that required electrical cardioversion to terminate. Intravenous administration of amiodarone was initiated, and this effectively suppressed the tachycardia. However, AF occurred on day 3, which immediately led to cardiogenic shock. Because AF was refractory to amiodarone, beta-blockers, and a total of 15 electrical cardioversions, the patient underwent radiofrequency catheter ablation on day 4 (Figure 1). Written informed consent was obtained from the patient’s spouse.

**Figure 2:**
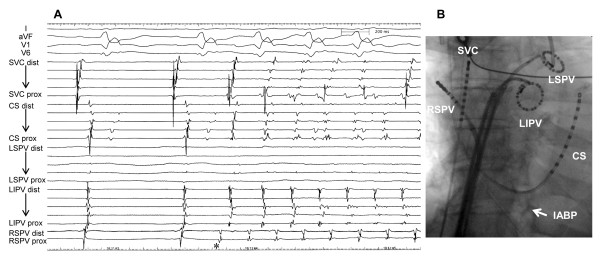
**A: The spontaneous onset of atrial fibrillation triggered by ectopies from the right superior pulmonary vein.** * The potential of right superior pulmonary vein preceded P wave by 63 ms. **B**: Fluoroscopic AP image demonstrating positions of the electrode catheters. The patient was on intra-aortic balloon pump during the procedure due to severe congestive heart failure. CS: coronary sinus; IABP = intraaortic balloon pump; LSPV = left superior pulmonary vein; LIPV = left inferior pulmonary vein; RSPV = right superior pulmonary vein; SVC = superior vena cava; dist = distal bipole; prox = proximal bipole.

**Figure 3:**
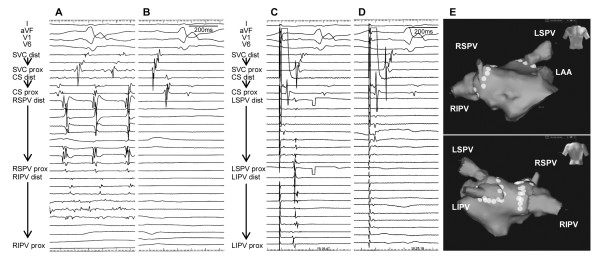
**Pulmonary vein isolation.A**: Baseline intracardiac electrograms of the right pulmonary veins before isolation during atrial fibrillation. **B**: Intracardiac electrograms of the right pulmonary veins after isolation. Sinus rhythm was restored by an electrical cardioversion following isolation of the right pulmonary veins. **C**: Baseline intracardiac electrograms of the left pulmonary veins before isolation during pacing from the distal bipoles of coronary sinus. **D**: Intracardiac electrograms of the left pulmonary veins after isolation. **E**: Three-dimensional map (NavX, St. Jude Medical) with white dots representing RF ablation lesions. LAA = left atrial appendage; RIPV = right inferior pulmonary vein. Other abbreviations as in Figure 2.

Prior to electrophysiological evaluation, the patency of the stented coronary artery was confirmed by coronary angiography. A decapolar catheter was positioned in the coronary sinus for pacing and used as a reference for the NavX (St. Jude Medical, St Paul, MN) system. Transseptal access was obtained using the standard Brokenbrough needle technique with intracardiac ultrasound and fluoroscopic guidance. Three 8 F SL0 sheaths were placed in the LA. Following pulmonary vein (PV) venography, the cardiac geometry was created by a 20-pole circular mapping catheter in all PVs, the LA appendage and the body of the LA. In order to identify the triggering vein, four electrode catheters were positioned in the following thoracic veins: a decapolar catheter in the superior vena cava, an 8-mm tip ablation catheter (Ablaze, Japan Lifeline, Japan) in the right superior PV, and two circular mapping catheters in the left superior PV and the left inferior PV, respectively. Shortly after electrical cardioversion, ectopies from the right superior PV triggered the initiation of AF (Figure 2). This phenomenon was reproducibly observed with the circular mapping catheters repositioned in the right superior PV and right inferior PV, respectively. Accordingly, the right PVs were circumferentially isolated at antrum during AF with the 8-mm tip ablation catheter at a power of 30–35 W under the guidance of a NavX 3D navigation system. Again, AF was electrically cardioverted, and sinus rhythm was restored (Figure 3). Subsequently, the left PVs were circumferentially isolated from LA (Figure 3). The absence of bidirectional PV-LA dormant conduction was confirmed by intravenous injection of 40 mg of adenosine. The stabilization of the hemodynamics was successfully achieved with an increase in the blood pressure and urine volume (Figure 1). CHDF and amiodarone were discontinued on days 5 and 6, respectively. Weaning from mechanical ventilation and intravenous dopamine was also initiated. The patient was free from AF recurrence until he suddenly died due to ventricular fibrillation on day 9.

## Discussion

To our knowledge, this is the first report of PV isolation (PVI) for a rescue purpose applied in a patient with hemodynamically unstable AF complicated with an AMI. The present case includes two major findings. First, AF may be triggered by ectopies from the PVs in the critical setting of AMI. Second, PVI may be effective for rhythm control and may thereby improve hemodynamic status.

AF, especially a new onset of AF, has been shown to be an independent predictor of mortality in patients with AMI [1,2]. AF may cause adverse hemodynamic effects, such as loss of atrial contraction, rapid ventricular rates, loss of atrioventricular synchrony, and an irregular RR interval, leading to a decrease in cardiac output [3]. Thus, when the patients are complicated with congestive heart failure, the development of AF should have greater adverse clinical significance than in cases without heart failure [4]. Systemic circulation may collapse immediately. Even if it does not, insufficient coronary flow due to decreased cardiac output may lead not only to delaying the myocardial healing process but also to further myocardial damage, which starts a vicious cycle of hemodynamic deterioration. In this setting, AF could be a lethal arrhythmia. The present case is a typical example. The hemodynamics were barely maintained during sinus rhythm with the mechanical support of an IABP, but they immediately deteriorated due to the development of AF.

Despite the increasing evidence showing the prognostic significance of AF in patients with AMI, current therapeutic strategies in this setting seem to be limited to the management of hemodynamics, including IABP, mechanical ventilation, CHDF, and so forth. In addition, sedation with opioid analgesic drugs such as sufentanil might provide some cardioprotective / antiarrhythmic effects. These are the therapies generally given to AMI patients with congestive heart failure, but are not a specific treatment for AF. The rate control strategy may be accepted as an alternative to sinus rhythm restoration. However, attempts at rate control often fail when patients are on high levels of endogenous or exogenous catecholamines or are not able to tolerate the use of drugs including beta-blockers, digoxin, and calcium antagonists, because these agents have negative inotropic effects or may increase oxygen consumption. Furthermore, sinus rhythm should be necessary to maintain systemic circulation in patients with severe pump failure as shown in the present case. Amiodarone might be a drug that can be used in this setting [4]. However, observational data recently suggested that an amiodarone-based rhythm control strategy in patients with AF after AMI complicated by heart failure or LV dysfunction is associated with excess early mortality when compared with the rate control strategy [5].

PVI has been accepted by consensus as the strategy of choice for the treatment of AF by catheter ablation [6]. PVI exerts its beneficial effects by autonomic denervation of LA, by eliminating part of the arrhythmogenic substrate, and most importantly by eliminating AF triggers arising from PVs [6,7]. However, it is not clear whether PVI also functions well in the setting of AMI where multiple precipitating factors may be involved in the development of AF. Those factors may include inflammation, acute hypoxia or hypokalaemia, endogenous or exogenous catecholamines, right ventricular infarction, and atrial ischemia [8]. Hemodynamic impairment secondary to LV dysfunction such as a high pulmonary artery wedge pressure as well as right atrial pressure relate to the development of AF [9]. The present case demonstrated that PVs play an important role as a source of AF triggers in this setting. PV-stretch due to elevated PV pressure might contribute to the triggering substrate [10]. Although the patient died due to ventricular fibrillation, PVI successfully brought the patient back into sinus rhythm and clearly improved his hemodynamic status.

Based on this single case report, it is premature to draw conclusions that PVI could become a standard procedure to treat AF in the critical setting of an AMI. Since PVI is still a highly complex procedure, a careful assessment of benefit and risk must be considered for each patient.

## Conclusions

Ectopic activity in the PVs may be responsible for triggering AF in the critical setting of an AMI. PVI in desperate cases, as a last resort, is feasible and could be an effective therapeutic option.

## Consent

Written informed consent was obtained from the patient’s spouse for publication of this case report and accompanying images.

## Abbreviations

AF: Atrial fibrillation; AMI: Acute myocardial infarction; CHDF: Continuous hemodiafiltration; IABP: Intraaortic balloon pump; LA: Left atrium; LV: Left ventricle; PV: Pulmonary vein; PVI: Pulmonary vein isolation.

## Competing interests

The authors declare that they have no competing interests.

## Authors’ contribution

IM (author of correspondence) performed pulmonary vein isolation and drafted the manuscript. TS gave final approval of the version to be published. HT and HM were involved in clinical decision making and in revising the manuscript critically for important intellectual content. All authors read and approved the final manuscript.

## Pre-publication history

The pre-publication history for this paper can be accessed here:

http://www.biomedcentral.com/1471-2261/12/110/prepub
